# Guy's Stone Score (GSS) Based on Intravenous Pyelogram (IVP) Findings Predicting Upper Pole Access Percutaneous Nephrolithotomy (PCNL) Outcomes

**DOI:** 10.1155/2016/5157930

**Published:** 2016-11-24

**Authors:** Bannakij Lojanapiwat, Pattara Rod-Ong, Pruit Kitirattrakarn, Wilaiwan Chongruksut

**Affiliations:** Division of Urology, Department of Surgery, Faculty of Medicine, Chiang Mai University, Chiang Mai, Thailand

## Abstract

*Objective.* To predict the success rate and complications following percutaneous nephrolithotomy via the upper pole using the Guy's Stone Score (GSS) based on the findings of a preoperative intravenous pyelogram (IVP).* Patients and Methods*. Two hundred and twenty-seven renal operations, which were carried out using PCNL via the upper pole, were classified according to the GSS assigned. Any complications were classified according to the Clavien classification. The success rates and incidence of any complications were compared between each GSS.* Results*. The immediate success rates were 87.50% of GSS1, 71.43% of GSS2, 53.62% of GSS3, and 38.46% of GSS4, *P* < 0.01. There were statistically significant differences between the groups in stone size, overall immediate success rate, operative time, number of access tracts, and frequency of tubeless PCNL. Major complications (a Clavien score of 3–5) were significantly higher in the cases with a higher GSS.* Conclusion*. A GSS based on an IVP is a simple and reliable tool in predicting the success rate and possible complications following upper pole access PCNL.

## 1. Introduction

Percutaneous nephrolithotomy (PCNL) is accepted as being the first line treatment for large renal and upper ureteric stones. The advantage of upper pole access for percutaneous nephrolithotomy is good stone clearance due to its direct access to most intrarenal collecting systems [1, 2]. Upper pole access can be achieved via both supracostal and subcostal routes. Due to the relative anatomic position of the upper pole of the kidney to the diaphragm and pleura, pulmonary complications are more common when this technique is used, especially of supracostal access [1, 2]. Upper pole access PCNL should provide the same success rate in all stone patients but may experience more pulmonary complication.

Despite advances in technology and surgical techniques, in complex stone cases, the PCNL technique does not always result in the optimal goal of stone free status and increases the risk of significant complications. The information about the success rate and complications following this procedure is very important for both surgeons and patients in informing surgical planning and preoperative patient advice and counseling. At the current time, the Guy's Stone Scoring (GSS) system [3–7], the STONE nephrolithometry scoring system [8, 9], and the CROES (Clinical Research Office of Endourological Society) nomogram [10] are used for the prediction of the success rate and possible complications following PCNL in research and clinical practice. Previous studies reported that the Guy's stone score is significantly associated with stone-free status [3–7]; however other studies reported an association of this score with complications [4, 6]. STONE nephrometric scores were significantly associated with stone free status but not with postoperative complications [8, 9]. Most previous studies of GSS in PCNL treatment were performed in general PCNL patients [3–7].

This study focusses on the association of GSS based on an intravenous pyelogram (IVP) and on success rate and complications following PCNL via both supracostal and subcostal upper pole access.

## 2. Patients and Methods

This study was approved for conduct clinical research from research committee of faculty of medicine, Chiang Mai University.

### 2.1. Patients

Two hundred and twenty-seven patients who underwent PCNL via upper pole access were recruited, including 146 subcostal access and 81 supracostal access cases. All patients with both kidney, ureter, bladder (KUB) film, and intravenous pyelogram (IVP) preoperatively were classified into 4 grades according to their Guy Stone Score (GSS):

- Guy's stone score 1 (GSS1): a solitary stone in the mid/and or lower pole or in the renal pelvis with a normal anatomy and simple collecting system
- Guy's stone score 2 (GSS2): a solitary stone in the upper pole; multiple stones in patients with simple anatomy; or a solitary stone in a patient with abnormal anatomy
- Guy's stone score 3 (GSS3): multiple stones in a patient with abnormal anatomy or in a calyceal diverticulum or partial staghorn calculus
- Guy's stone score 4 (GSS4): a complete staghorn calculus or any stone in a patient with spinal bifida or a spinal injury, calculus in patients with clinical neurological alternations (spinal cord injury, myelomeningocele)

Of the total 227 patients, the mean age was 54.28 ± 13.34, 51.77 ± 10.07, 55.12 ± 0.92, and 55.51 ± 10.03 years in GSS1, GSS2, GSS3, and GSS4, respectively. In the subgroup of patients who underwent supracostal access, the mean ages were 51.00 ± 18.14, 53.00 ± 10.68, 51.52 ± 9.17, and 53.24 ± 9.29 years, respectively.

### 2.2. Methods

All procedures were carried out following the evaluation of hemostasis, renal function, and anatomy of collecting system by conventional serum laboratory techniques and intravenous pyelography. General anesthesia was administered. In the supine position, an open ended 6 Fr ureteral catheter was placed transurethrally into the upper ureter or renal pelvis. All punctures were performed in the prone position at the upper pole (81 supracostal, 146 subcostal) after the injection of contrast media via the ureteric catheter under fluoroscopic guidance. All supracostal access was performed between the 11th and 12th rib into the lower part of the intercostal space with close coordination with the anesthesiologist for respiration control. The puncture was made through the retroperitoneum during full expiration, and the needle passed through the renal parenchyma into the collecting system during deep inspiration. Both working and safety guide wires were inserted after the needle was in collecting system. Tract dilatation was performed using an Amplatz dilator (Cook Urologic, Spencer, IN) or telescopic metal dilators size 8 Fr to 30 Fr followed by the Amplatz sheath 30 Fr. A standard 26 Fr nephroscope was used utilizing ultrasonic or pneumatic lithotripsy for stone disintegration. The collecting system was examined using fluoroscopy and direct inspection using a nephroscope at the end of the procedure. A 20 Fr nephrostomy tube was inserted for 48–72 hours. No nephrostomy tube was inserted if there was no significant bleeding, no significant extravasation, no distal obstruction, and no retained stone (tubeless PCNL). Postoperative chest radiographs were obtained in all cases for the evaluation and treatment of any possible pulmonary complications in recovery room.

### 2.3. PCNL Outcome

The immediate success rate was defined as the patient being stone-free or the presence of asymptomatic fragments of less than or equal to 4 mm in plain KUB film postoperative day 1. Complications were graded according to the Clavien score system, which is divided into 5 grades. Minor complications were defined as Clavien score grades 1-2 and major complications were defined as Clavien 3–5.

### 2.4. Statistical Analysis

Statistical analysis using ANOVA Prosthoc and Fisher's exact test was performed, and *P* < 0.05 was considered statistically significant.

## 3. Results

### 3.1. Total Patients (*N* = 277)

The average stone size was 27.03 ± 0.60 mm in GSS1, 26.42 ± 10.09 mm in GSS2, 42.28 ± 11.79 mm in GSS3, and 63.24 ± 17.28 mm in GSS4, *P* < 0.01. The operative time was 48.21 ± 21.70 min in GSS1, 65.50 ± 23.80 min in GSS2, 68.91 ± 29.43 min in GSS3, and 76.30 ± 33.40 min in GSS4, *P* < 0.01. Tubeless PCNL was 18.75%, 20.0%, 8.7%, and 5.49%, in GSS1, GSS2, GSS3, and GSS4, respectively (*P* = 0.03). The immediate success rate was 87.50% in GSS1, 71.43% in GSS2, 53.62% in GSS3, and 38.46% in GSS4, *P* < 0.01. Major complications (Clavien 3–5) were 3.13%, 17.14%, 30.43%, and 29.67% in GSS1, GSS2, GSS3, and GSS4, respectively, *P* < 0.01. There was no significant difference in age, number of incidents of previous open surgery, number of access tracts, and blood transfusion between each GSS. The demographic data with the results is shown in Tables 1 and 2.

### 3.2. Supracostal Access Patients (*N* = 81)

The average stone size was 24.16 ± 3.43 mm in GSS1, 27.00 ± 9.15 mm in GSS2, 43.27 ± 11.91 mm in GSS3, and 61.97 ± 20.04 mm in GSS4, *P* < 0.01. The immediate success rate was 83.33% in GSS1, 66.66% in GSS2, 64.00% in GSS3, and 34.14% in GSS4, *P* < 0.01. Major complications (Clavien 3–5) were 0% in GSS1 and GSS2, 40% in GSS3, and 31.71% in GSS4, *P* = 0.03. There was no significant difference in age, number of incidents of previous open surgery, number of access tracts, operative time, and rate of tubeless and blood transfusion between each GSS. The demographic data with the results is shown in Tables 3 and 4.

## 4. Discussion

PCNL via the upper pole was shown to achieve a greater stone free rate due to this technique being able to reach most of the intrarenal collecting system especially in the treatment of complex stones. The incidence of pulmonary complication is significantly higher in PCNL via upper pole access especially when access is the supracostal route.

Several factors are associated with post-PCNL outcomes including stone size, stone burden, degree of hydronephrosis, history of previous open nephrolithotomy, and anomalies of the kidney [11–14]. de la Rosette et al. reported the increase of complications when PCNL was performed in patients who had larger stones [15]. In contrast, Olbert et al. found that the increase in the size of the stone did not relate to postoperative stone free status and complication rate but did relate to operative time and length of hospital stay [16]. Turna et al. reported that only stone composition was related to blood loss [12]. Penbegul et al. report the same outcomes following PCNL in patients with or without abnormal renal anatomy [13, 14].

Previously, there was no definite tool to predict PCNL outcome and rate of complication, which is very important to both physicians and patients. The tools should indicate the difficulty or risk related to the surgery, which is the information required for patient counseling and surgeon communication. Several studies have attempted to classify PCNL to predict the outcomes [11, 12, 16, 17] and complications [16–20]. Most of these studies did not result in any consistent correlation as regards predictions and none of these are in common use in clinical practice. A quick, simple, and reproducible tool is needed in real life practice to predict outcomes and complications following PCNL. This is very important for patient counseling, surgeon training, and service planning.

Recently, urolithiasis scoring systems for percutaneous nephrolithotomy outcome have been developed including the Guy Stone Score (GSS), STONE nephrometry and the CROES nephrolithometric nomogram. The Guy Stone Score, STONE nephrometry, and CROES nomogram serve as disease stratification tools for surgical planning and patient counseling in aspects of surgical outcome, but only Guy's Score and STONE nephrometry are associated with possible complications.

Guy Stone Score (GSS) is a simple and reliable tool for predicting success rate. GSS is mainly used in kidney, ureter, bladder (KUB) film, and intravenous urography (IVU) to predict the success rate following PCNL. Some studies reported the usage of computed tomographic scan in GSS estimation for more accuracy. The CT scan was used as the preoperative investigation in stone patients, which improved the accuracy of stone information and renal and collecting system anatomy [4, 5]. Vicentini and colleagues used Guy's Stone Score (GSS) to predict percutaneous nephrolithotomy outcomes in the supine position based on preoperative computed tomographic scan in 155 renal cases. They confirmed the usefulness of the GSS tool based on CT findings in the accuracy of evaluation of renal stones in respect of surgical outcome and complications [4].

We agree that a preoperative CAT scan provided more accuracy regarding the detail of the anatomy such as characterization of the stone and pelvicalyceal anatomy, which are the factors in scoring these tools. But, GSS with plain KUB and intravenous urography is inexpensive and is a common routine investigation in stone patients especially in developing countries with a high prevalence of stone disease. These investigations have the additional advantage of providing a lower radiation dose than a CT scan. This is the initial study reporting on the use of GSS based on IVP in prediction regarding the outcome of PCNL with upper pole access, either supracostal or subcostal access.

This study confirmed that GSS based on KUB and intravenous urography is a valuable tool in the prediction of the outcome and complication rate following PCNL via the upper pole. Immediate success rates, operative time, tubeless rates (rate of uncomplicated procedure), and major complications are significantly different within the group of each GSS. There was few patients in this study who need multiple tracts and received blood transfusion. Fewer numbers of all positive outcome parameters and more major complications were found in higher GSS patients. A preoperative CT scan is not a common investigation in developing countries. The information in grading patients of GSS can be achieved from plain KUB and intravenous urography due to its simple classification in routine clinical practice. This nomogram does not need complex calculations. Our study demonstrated that plain KUB and intravenous urography are as useful in informing the classification grading of the GSS score as the CT scan. The results of this study demonstrates the correlation of the GSS grade with post-upper pole access PCNL outcome and complications in a large number of patients.

*The Limitations.* The number of patients in this study is small especially with supracostal access in GSS1 and GSS2. This limitation can be explained by the less common use of supracostal access in patients with lower GSS. A future larger prospective or meta-analysis study is needed. This study did not recruit patients with other factors such as kidney anomalies that may affect the outcome. Only Guy's Stone Score (GSS) was utilized in this study based on an intravenous pyelogram (IVP) to predict the outcome, so it cannot be compared with other Stone Scores, which required a CAT scan.

## 5. Conclusion

Guy's Stone Score (GSS) based on interpretation of an intravenous pyelogram is a simple and reliable tool in predicting the immediate success rate and possible complications following supracostal and subcostal upper pole access PCNL. This nomogram is very helpful in preoperative patient counseling and informing the decision about referral of more complicated patients to centers with higher patient volume and more expertise.

## Figures and Tables

**Table 1:** The demographic data of total upper pole access patients (*N* = 227).

	GSS1	GSS2	GSS3	GSS4	*P* value
	*N* = 32	*N* = 35	*N* = 69	*N* = 91	
Age (year)					
Mean (SD)	54.28 (13.34)	51.77 (10.07)	55.52 (0.961)	55.51 (10.03)	0.13
Gender, *n* (%)					
Male : female	12 : 20	21 : 14	48 : 21	62 : 29	0.01
Previous surgery, *n* (%)	10 (31.25)	10 (28.57)	16 (23.19)	22 (24.18)	0.79
Stone side, (*N*)					
Right : left	17 : 15	15 : 20	37 : 32	50 : 41	0.67
Size (mm):					
Mean (SD)	27.03 (0.60)	26.42 (10.09)	42.28 (11.79)	63.24 (17.28)	<0.01
BMI, *n* (%)					
18–25 cm/kg^2^	33 (66.34)	19 (63.33)	42 (73.22)	35 (76.08)	0.75
>25 cm/kg^2^	16 (32.65)	11 (36.67)	15 (26.79)	11 (23.91)	
Comorbidities					
Diabetes, *n* (%)	4 (13.79)	7 (21.88)	10 (20.83)	11 (16.92)	0.81
Hypertension, *n* (%)	12 (41.38)	11 (34.38)	22 (45.83)	33 (50.77)	0.47
Dyslipidemia, *n* (%)	7 (24.14)	6 (18.75)	8 (16.67)	15 (23.08)	0.81
Blood Cr., *n* (%)					
<1.5 mg%	43 (78.18)	23 (74.19)	53 (88.33)	40 (74.07)	0.22
>1.5 mg%	12 (21.82)	8 (25.81)	7 (11.67)	14 (25.93)	

**Table 2:** Outcomes of total upper pole access operations (*N* = 227).

	GSS1	GSS2	GSS3	GSS4	*P* value
	*N* = 32	*N* = 35	*N* = 69	*N* = 91	
Number of access tracts, *n* (%)					
Mean (SD)					
1	31 (96.88)	33 (94.29)	61 (88.41)	82 (90.11)	0.48
>1	1 (3.13)	2 (5.71)	8 (11.54)	9 (9.89)	
Op. time (min)					
mean (SD)	48.21 (21.70)	65.5 (23.80)	68.91 (24.43)	76.30 (33.44)	<0.01
Blood transfusion					
mean (SD)	1 (3.13)	3 (8.57)	4 (5.80)	4 (4.40)	0.76
Tubeless, *n* (%)	6 (18.75)	7 (20.00)	6 (8.70)	5 (5.49)	0.03
Immediate success					
*n* (%)	28 (87.50)	25 (71.43)	37 (53.62)	35 (38.46)	<0.01
Complications, *n* (%)					
Minor (Clavien 1-2)	3 (9.37)	14 (40.00)	21 (30.43)	25 (20.47)	<0.01
Major (Clavien 3–5)	0	2 (5.70)	7 (10.14)	13 (16.48)	<0.01
Sepsis	0	1	5	5	
Pulmonary complication	0	1	1	5	
Other	0	0	1	3	
Auxiliary treatment and					
re-PCNL, *n* (%)	2 (6.25)	1 (2.86)	18 (25.99)	38 (41.75)	0.02

**Table 3:** The demographic data of supracostal upper access patients (*N* = 81).

	GSS1	GSS2	GSS3	GSS4	*P* value
	*N* = 6	*N* = 9	*N* = 25	*N* = 41	
Age (year),					
Mean (SD)	51 (18.14)	53 (10.68)	51.52 (9.17)	53.24 (9.29)	0.11
Gender, (*N*)					
Male : female	1 : 5	8 : 1	16 : 9	32 : 9	0.01
Previous surgery, *N* (%)	1 (16.67)	1 (11.11)	5 (20.00)	11 (26.83)	0.81
Stone side, *N* (%)					
Right : left	31 : 25	14 : 18	29 : 34	27 : 29	0.72
Size (mm),					
Mean (SD)	24.16 (3.43)	7 (9.15)	43.27 (11.91)	61.97 (20.04)	<0.01
BMI					
18–25 cm/kg^2^	3 (50.00)	6 (75.00)	13 (68.42)	23 (65.76)	0.91
>25 cm/kg^2^	3 (50.00)	2 (25.00)	6 (31.58)	10 (31.25)	
Comorbidities					
Diabetes, *n* (%)	1 (16.67)	2 (25.00)	5 (33.33)	6 (20.00)	0.76
Hypertension, *n* (%)	3 (50.00)	2 (25.00)	6 (40.00)	15 (50.00)	0.62
Dyslipidemia, *n* (%)	2 (33.30)	1 (12.50)	2 (13.33)	7 (23.33)	0.67
Blood Cr.					
<1.5 mg%	5 (83.33)	5 (62.50)	17 (77.27)	26 (65.00)	0.63
>1.5 mg%	1 (16.67)	3 (37.50)	5 (22.73)	14 (35.00)	

**Table 4:** Outcomes of supracostal upper pole access operations (*N* = 81).

	GSS1	GSS2	GSS3	GSS4	*P* value
	*N* = 6	*N* = 9	*N* = 25	*N* = 41	
Operative time (min)					
Mean (SD)	50.57 (20.31)	67 (24.50)	68.46 (23.00)	78.71 (35.09)	0.17
Number of access tracts					
1	6 (100.00)	8 (88.89)	23 (92.00)	39 (95.12)	0.68
>1	0 (0.00)	1 (11.11)	2 (8.00)	2 (4.88)	
Immediate success rate *n* (%)	5 (83.33)	6 (66.66)	16 (64.0)	14 (34.14)	<0.01
Blood transfusion *n* (%)	1 (16.67)	1 (11.11)	2 (8.00)	2 (4.88)	0.41
Complications, *n* (%)					
Minor (Clavien 1-2)	0 (0)	4 (33.33)	8 (32.00)	5 (12.19)	
Major (Clavien 3–5)	0 (0)	1 (11.11)	5 (20.00)	12 (29.20)	0.03
Sepsis	0	0	2	4	
Pulmonary complication	0	1	1	6	
Other	0	0	2	2	
Auxiliary treatment and re-PCNL, *n* (%)	1 (16.67)	0 (0)	8 (32.00)	15 (36.58)	0.21
